# Design, Synthesis, and Biological Evaluation of a Small-Molecule PET Agent for Imaging PD-L1 Expression

**DOI:** 10.3390/ph16020213

**Published:** 2023-01-30

**Authors:** Liang Xu, Lixia Zhang, Beibei Liang, Shiyu Zhu, Gaochao Lv, Ling Qiu, Jianguo Lin

**Affiliations:** 1 School of Basic Medical Sciences, Wenzhou Medical University, Wenzhou 325035, China; 2 NHC Key Laboratory of Nuclear Medicine, Jiangsu Key Laboratory of Molecular Nuclear Medicine, Jiangsu Institute of Nuclear Medicine, Wuxi 214063, China

**Keywords:** PET, PD-L1, small-molecule imaging agent, tumor

## Abstract

Immunotherapy blocking programmed cell death protein 1/programmed death ligand 1 (PD-1/PD-L1) pathway has achieved great therapeutic effect in the clinic, but the overall response rate is not satisfactory. Early studies showed that response to treatment and overall survival could be positively related to PD-L1 expression in tumors. Therefore, accurate measurement of PD-L1 expression will help to screen cancer patients and improve the overall response rate. A small molecular positron emission tomography (PET) probe [^18^F]**LP**-**F** containing a biphenyl moiety was designed and synthesized for measurement of PD-L1 expression in tumors. The PET probe [^18^F]**LP**-**F** was obtained with a radiochemical yield of 12.72 ± 1.98%, a radiochemical purity of above 98% and molar activity of 18.8 GBq/μmol. [^18^F]**LP**-**F** had good stability in phosphate buffer saline (PBS) and mouse serum. In vitro assay indicated that [^18^F]**LP**-**F** showed moderate affinity to PD-L1. Micro-PET results showed that the tumor accumulation of [^18^F]**LP**-**F** in A375 tumor was inferior to that in A375-hPD-L1 tumor. All the results demonstrated that [^18^F]**LP**-**F** could specifically bind to PD-L1 and had a potential application in non-invasive evaluation of PD-L1 expression in tumors.

## 1. Introduction

Immunotherapy based on immune checkpoint blockade has developed greatly in the past decade for the treatment of cancers, opening a new era of oncotherapy. PD-L1 is PD-1’s major ligand, which can be overexpressed in a variety of malignancies [1]. Once PD-1 binds to PD-L1, not only can the PD-1/PD-L1 pathway inhibit the activation and physiological function of T cells, the proliferation of NK cells and the production of B cell antibodies, but can also promote the stability of Treg cells to enhance their inhibitory function, thus leading to immune suppression and tumor immune evasion [1,2]. The overall response rate of cancer patients followed by PD-1/PD-L1 blocked treatment is only 5–30% [3] in spite of the remarkable clinical results achieved. Some studies indicated that PD-L1-positive patients may have higher clinical therapy effectiveness and a longer median survival time after immunotherapy [4,5,6,7,8,9]. Therefore, accurate measurement of PD-L1 expression levels in tumors has important clinical guiding significance.

To date, immunohistochemistry (IHC) is still the commonly used approach to evaluate PD-L1 expression levels in the clinic. However, it has the following drawbacks. First, it is necessary to obtain tumor tissue by invasive procedures such as biopsy. Second, immunohistochemistry can only evaluate PD-L1 expression levels in pathological sections rather than reflect the whole body or metastasis [10]. Third, the expression level at tumor sites will be modulated by treatment administration, such as chemotherapy and radiotherapy [11,12,13], but IHC could not monitor it in real time. Some studies, however, have reported that there was no correlation between the immunotherapy response rate of patients receiving anti-PD-L1 immunotherapy and the PD-L1 expression level in tumors [14,15,16]. This may be caused by the intra-/inter-tumoral heterogeneity and the differences in the definition of PD-L1 positivity in IHC [17,18].

PET can non-invasive monitor mutations in PD-L1 expression levels in lesions in real time, dynamically and systemically, avoiding the defects of IHC. It provides a new strategy for determination of PD-L1 expression. At present, researchers have developed many PET imaging agents targeting PD-L1 based on monoclonal antibodies. For example, ^64^Cu-atezolizumab could accurately assess PD-L1 expression [19] and ^89^Zr-atezolizumab could predict clinical response to anti-PD-L1 blocking therapy [20]. However, antibody-based imaging agents need a long interval time between the injection and imaging to measure target expression owing to the long biological half-life [13,21]. Consequently, radionuclides with a longer half-life are demanded for labeling, resulting in high radiation dosimetry to body.

Compared with antibody-based imaging agents, small-molecule imaging agents have the advantages of low cost, good tumor penetration, fast tissue uptake and rapid imaging. For example, [^64^Cu/^68^Ga]DPA [22], D-dodecapeptide-based radiotracers, could image PD-L1 expression tumor with high a signal to noise ratio within 60 min. ^68^Ga-NOTA-WL12 based on peptide could also acquire tumor images rapidly with reasonable radiation dosimetry [23]. Therefore, small-molecule imaging agents have great potential to measure PD-L1 expression levels rapidly. Recently, many reports have demonstrated that small molecules containing biarylmethyl aryl ether scaffold showed high affinity to PD-L1 [24,25,26,27,28]. In our previous works, two PD-L1 PET probes based on the biarylmethyl aryl ether scaffold, [^18^F]**LN** and [^18^F]**LG**-**1**, have been designed and synthesized (Figure S16A) [29,30]. [^18^F]**LN** had poor water solubility and could not show the outline of PD-L1^+^ tumor clearly and defluorination restricted and hindered its further application (Figure S16B). As for [^18^F]**LG**-**1**, the solubility and tumor to muscle ratio (T/M) value has been improved greatly (Figure S16B), but it was necessary to take two steps of labeling to obtain the product.

**Scheme 1:**
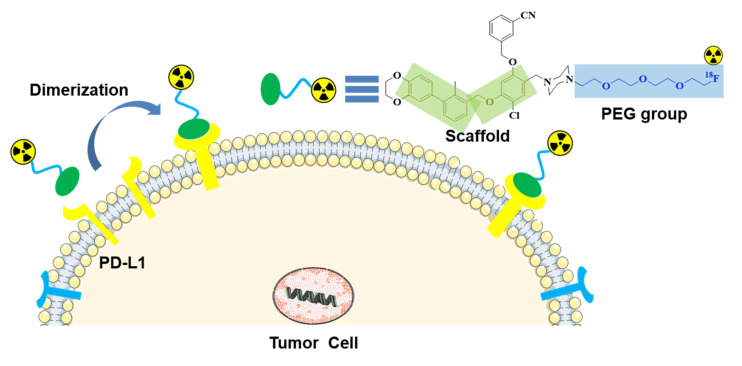
Design and chemical structure of [^18^F]**LP**-**F** for imaging PD-L1 expression.

Given this, a new small molecular radiotracer [^18^F]**LP**-**F** (Scheme 1) bearing the same scaffold as [^18^F]**LN** and [^18^F]**LG**-**1** was developed by simple one-step ^18^F-fluorination. A poly(ethylene glycol) (PEG) moiety was introduced to mediate the pharmacokinetics of the tracer including increased water solubility and reduced renal clearance, which had been widely used in development of new radiotracers [31,32,33]. Pharmacokinetics and in vivo behaviors of [^18^F]**LP**-**F** were studied by PET imaging. It was expected that [^18^F]**LP**-**F** could distinguish tumors with different target expression levels.

## 2. Results

### 2.1. Synthesis of Precursor LP2 and Non-Radioactive Probe LP-F

**Scheme 2:**
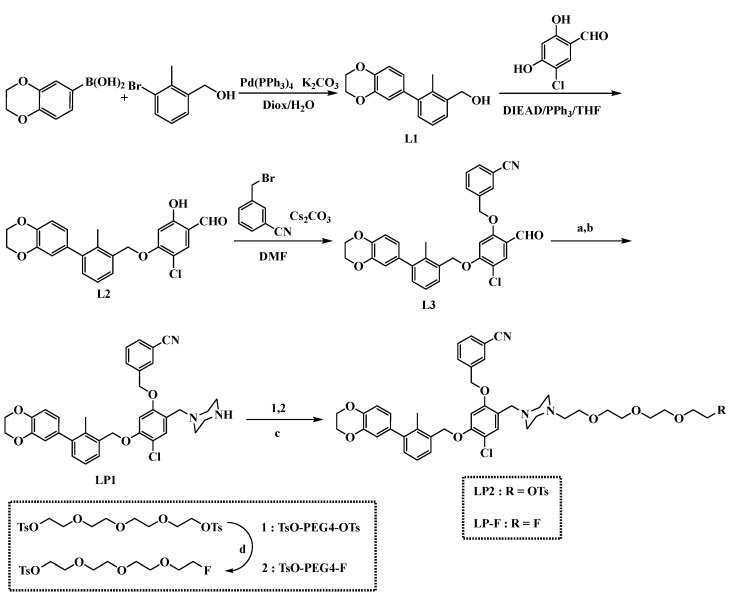
Synthesis of the precursor compound **LP2** and the non-radioactive probe **LP**-**F**. Reaction conditions: (**a**) piperazine, AcOH, DMF, 45 °C, 4.5 h; (**b**) NaBH_3_CN, 24 h; (**c**) appropriate toluenesulfonate, K_2_CO_3_, CAN, 85 °C; (**d**) TBAF, THF, 80 °C.

Compounds **LP2** and **LP**-**F** were prepared by the following reactions (Scheme 2). The synthesis of compounds **L1**–**L3** had been reported in our previous work [29]. Compound **L3** reacted with pyrazine through a reductive amination reaction to obtain compound **LP1**. Then, compound **LP1** reacted with **TsO-PEG4-OTs** catalyzed by potassium carbonate to afford the precursor compound **LP2**. Substitution of compound **TsO-PEG4-OTs** with tetrabutylamine fluoride (TBAF) afforded **TsO-PEG4-F**. Similarly, **LP**-**F** was synthesized through the nucleophilic substitution reaction between **LP1** and **TsO-PEG4-F**. Chemical structures of all the compounds were characterized by HPLC, ESI-MS and ^1^H/^13^C/^19^F NMR spectra (Figures S1–S13).

### 2.2. Radiochemistry

**Figure 1:**
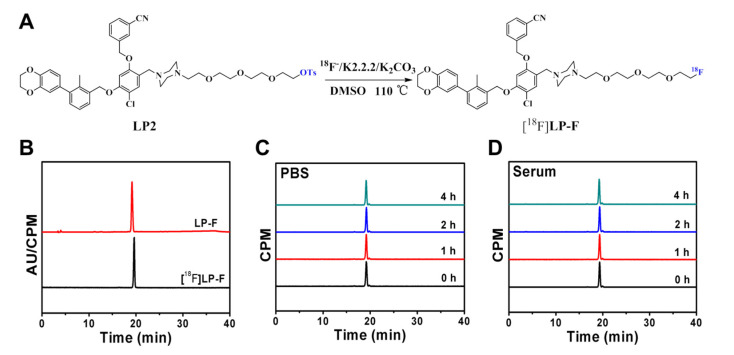
Radiosynthesis and HPLC characterization of the tracer [^18^F]**LP**-**F**. (**A**) Radiosynthesis of [^18^F]**LP**-**F** via nucleophilic substitution reaction. (**B**) HPLC analysis of [^18^F]**LP**-**F** (radio) and **LP**-**F** (UV). (**C**,**D**) The stability of [^18^F]**LP**-**F** in PBS (**C**) and mouse serum (**D**) at 37 °C measured by radio-HPLC.

[^18^F]**LP**-**F** was obtained simply by direct ^18^F-fluorination of the precursor **LP2** with kryptofix 2.2.2 and K_2_CO_3_ at 110 °C in DMSO (Figure 1A). The same retention time of [^18^F]**LP**-**F** (19.3 min) and **LP**-**F** (19.2 min) indicated the successful radiosynthesis (Figure 1B). The radio-conversion was over 70% and the final radiochemical yield was 12.72 ± 1.98% after purification by pre-HPLC with the radiochemical purity more than 98% (Figure 1B). The molar activity of [^18^F]**LP**-**F** was estimated to be 18.8 GBq/μmol (Figure S15). The partition coefficient (Log *P*) of [^18^F]**LP**-**F** was calculated to be 2.18 ± 0.16 (Table S2). [^18^F]**LP**-**F** showed high stability in PBS and mouse serum in 4 h as shown in Figure 1C,D.

### 2.3. Cell Uptake and Specific Binding

**Figure 2:**
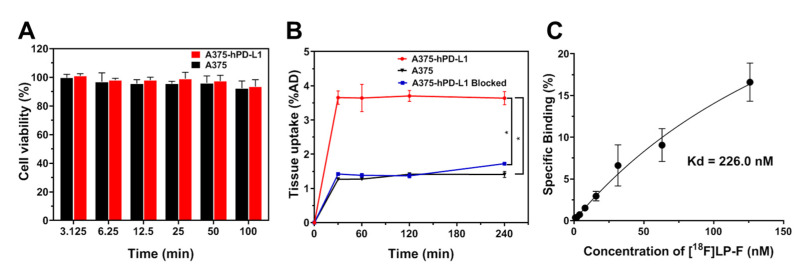
(**A**) Cell viability of PD-L1^-^ and PD-L1^+^ cells incubated with **LP**-**F** for 24 h. (**B**) The cellular uptake of [^18^F]**LP**-**F** in A375 cells, A375-hPD-L1 cells and A375-hPD-L1 cells blocked by **LP**-**F**. (**C**) Specific binding of [^18^F]**LP**-**F** to PD-L1 in PD-L1^+^ cell in vitro. * *p* < 0.05.

The MTT assay was performed in PD-L1^+^ (A375-hPD-L1) cells and PD-L1^-^ (A375) cells to investigate the cytotoxicity of **LP**-**F**. The cell viability of all treated tumor cells was over 90% (Figure 2A). These results demonstrated the low cytotoxicity and satisfactory biocompatibility of **LP**-**F**. The cell uptake of [^18^F]**LP**-**F** in PD-L1^+^ and PD-L1^−^ tumor cells was studied to evaluate the target of [^18^F]**LP**-**F** to PD-L1-positive tumor cells. The accumulation of [^18^F]**LP**-**F** in PD-L1^+^ cells quickly reached 3.66 ± 0.20 %AD at 30 min, and remained stable at 3.64 ± 0.19 %AD at 4 h as shown in Figure 2B. However, for PD-L1^-^ cells, the uptake of [^18^F]**LP**-**F** was always at a low level. The maximum uptake value was determined to be 1.41 ± 0.09 %AD at 4 h. After blockade by **LP**-**F**, the uptake of [^18^F]**LP**-**F** in PD-L1^+^ cells obviously decreased to 1.42 ± 0.03 %AD at 0.5 h and 1.72 ± 0.02 %AD at 4 h, which was compared to that of PD-L1^-^ cells. The dissociation constant (K_d_) value of [^18^F]**LP**-**F** to the PD-L1^+^ cell was measured to be 226.0 nM by the saturation binding assay (Figure 2C).

### 2.4. Pharmacokinetics

**Figure 3:**
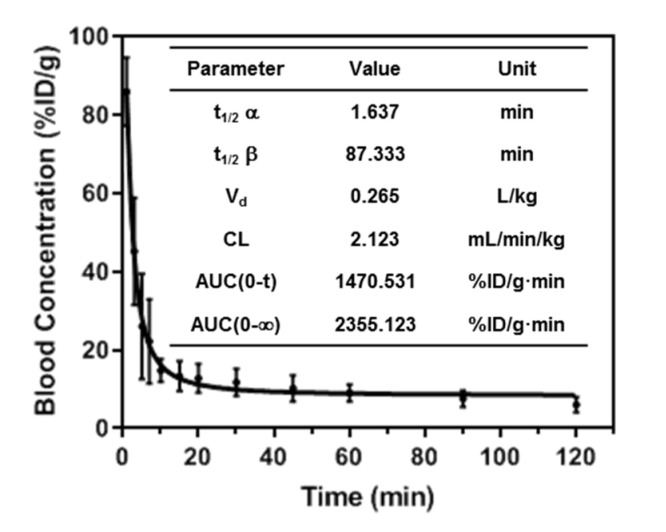
The drug concentration–time profiles and pharmacokinetic parameters of [^18^F]**LP**-**F** in normal mice (*n* = 3) within 2 h.

The pharmacokinetics was an important parameter for PET tracers, and small molecule-based probes always got benefits from the optimum pharmacokinetic characteristics. Thus, the pharmacokinetics of [^18^F]**LP**-**F** was studied as the metabolism of radioactive dose in blood. As shown in Figure 3, the activity in blood decreased rapidly within 10 min and then eliminated slowly. After data fitting in DAS 2.1 software using a two-compartment model, the half-life of distribution was calculated to be approximately 1.64 min and the half-life of elimination was approximately 87.33 min. Additionally, the apparent distribution volume of [^18^F]**LP**-**F** was 0.265 L/kg.

### 2.5. Micro-PET Imaging

**Figure 4:**
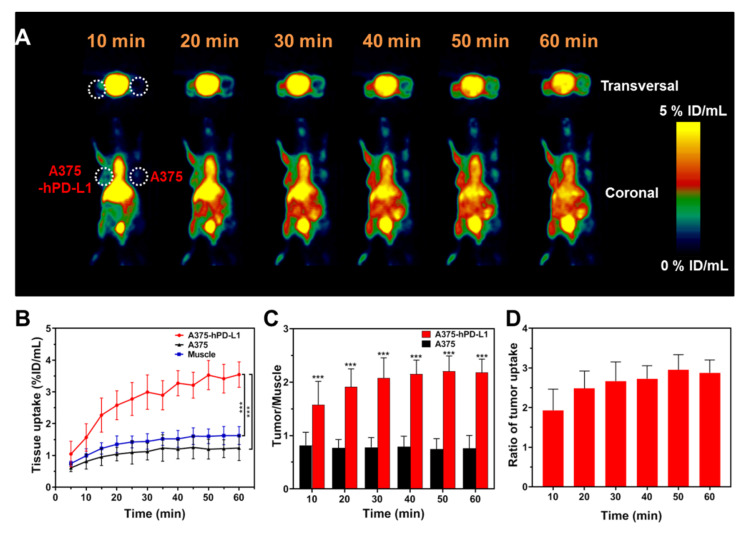
In vivo micro-PET imaging in A375 (right) and A375-hPD-L1 (left) bilateral tumor-bearing mice (*n* = 3). (**A**) Transversal and coronal images of PD-L1^-^/ PD-L1^+^ tumor-bearing mice from 10 to 60 min. (**B**) Tissue uptake of [^18^F]**LP**-**F** in tumors and muscle. (**C**) The tumor/muscle ratios of PD-L1^-^ and PD-L1^+^ tumor. (**D**) The ratio of PD-L1^+^ tumor uptake to PD-L1^-^ tumor uptake. *** *p* < 0.001.

Micro-PET imaging was performed in A375 (right) and A375-hPD-L1 (left) bilateral tumor-bearing nude mice. The representative dynamic images were obtained as shown in Figure 4A. [^18^F]**LP**-**F** could show the outline of A375-hPD-L1 tumor more clearly than that of A375 tumor. The accumulation of [^18^F]**LP**-**F** in PD-L1^+^ tumor increased constantly and reached to the maximum 3.53 ± 0.46 %ID/mL at 50 min (Figure 4B) while the activity in PD-L1^-^ tumor was at a low level and always weaker than that in PD-L1^+^ tumor at any time. The maximum was observed to be 1.23 ± 0.39 %ID/mL at 60 min (Figure 4B). [^18^F]**LP**-**F** showed a low uptake in muscle from 0.75 to 1.62 %ID/mL within the whole measuring process. The tumor-to-muscle ratio for PD-L1^+^ tumor was calculated to be 2.08 ± 0.38 post injection of [^18^F]**LP**-**F** at 30 min, and increased to 2.20 ± 0.29 at 50 min (Figure 4C). However, the value of T/M for PD-L1^-^ tumor was below 1 all the time and ranged from 0.75 ± 0.19 at 50 min to 0.82 ± 0.24 at 10 min (Figure 4C). In Figure 4D, the uptake of [^18^F]**LP**-**F** in PD-L1^+^ tumor was 2.95 ± 0.39 times higher than that in PD-L1^-^ tumor at 50 min. Notably, there was no obvious activity on the bone indicating the non-defluorination and good in vivo stability of [^18^F]**LP**-**F**. All the results confirmed that [^18^F]**LP**-**F** could make a distinction between tumors with various PD-L1 expression levels.

## 3. Discussion

Immunotherapy of blockage of the PD-1/PD-L1 interaction has made great achievements in the clinic. However, it is particularly crucial to improve the response rate. PD-L1 in tumors was the most studied biomarker to predict clinical effectiveness [34]. PET provides a new strategy to detect PD-L1 expression. Our group has designed two small-molecule radiotracers based on the biarylmethyl aryl ether scaffold, [^18^F]**LN** and [^18^F]**LG**-**1**. Both of them showed specific binding to PD-L1. However, the defluorination of [^18^F]**LN** and complicated two-step radiosynthesis of [^18^F]**LG**-**1** promoted us to further improve the design of the tracers. In this work, [^18^F]**LP**-**F** was designed by introducing a PEG group to the same scaffold to optimize the pharmacokinetics. Furthermore, [^18^F]**LP**-**F** could be obtained simply by one-step radiosynthesis via nucleophilic substitution of the introduced OTs group with a high radio-converting ratio. It showed high in vitro stability as proved by the radio-HPLC after incubation in PBS and mouse serum, and high in vivo stability since no obvious defluorination was observed in PET imaging. **LP**-**F** possessed more satisfactory biocompatibility than **LN** and **LG**-**1**.

The significant difference (*p* < 0.05) of the cellular uptake between the PD-L1^+^ and PD-L1^-^ cells demonstrated that [^18^F]**LP**-**F** could specifically accumulate in high PD-L1 expression cells. The high uptake of [^18^F]**LP**-**F** at 30 min in PD-L1^+^ cells demonstrated that [^18^F]**LP**-**F** could enter cells rapidly. Additionally, the non-radioactive probe **LP**-**F** could significantly inhibited the uptake of [^18^F]**LP**-**F** in PD-L1^+^ tumor cells, further indicating the specificity of [^18^F]**LP**-**F** to PD-L1.

The dissociation constant (Kd) value of [^18^F]**LP**-**F** was 226.0 nM, inferior to that of [^18^F]**LN** (65.27 nM) and [^18^F]**LG**-**1** (63.13 nM), indicating a little weaker affinity of [^18^F]**LP**-**F**. Differences in affinity exhibited were due to the PEG group in the solvent interaction region. It has been reported that such small inhibitors containing more hydrogen-bond donors in the solvent interaction region, such as –OH, -NH_2_ and –COOH, had better solubility in water and formed H-bindings with homodimeric PD-L1, thus exhibiting higher affinity [26,27,28]. Yet, the PEG group containing hydrogen bond receptor rather than hydrogen-bond donor in the solvent interaction region may not contribute to the affinity of [^18^F]**LP**-**F** to PD-L1 though pegylation can improve solubility of the scaffold.

The results of pharmacokinetics showed the rapid distribution and slow elimination of [^18^F]**LP**-**F** and it was eliminated as first-order kinetics. The half-life of elimination of [^18^F]**LP**-**F** matched the decay half-life (109.8 min) of fluorine-18, which meant low radiation risk to body. The apparent distribution volume of [^18^F]**LP**-**F** was 0.265 L/kg. Therefore, it was mainly distributed into tissues rather than blood pool, which was also confirmed by PET imaging.

Micro-PET imaging results showed that [^18^F]**LP**-**F** could selectively accumulate in high PD-L1 expression tumor. It could rapidly enter tumors within 30 min and showed sustained relatively higher T/M values in PD-L1^+^ tumor than that in PD-L1^-^ tumor. Thus, PET imaging of [^18^F]**LP**-**F** could clearly distinguish between the high and low PD-L1 expression tumors. [^18^F]**LP**-**F** showed higher tumor uptake than of [^18^F]**LN**, but lower than of [^18^F]**LG**-**1**. As mentioned above, the introduction of PEG chain improved the pharmacokinetics and bioavailability of [^18^F]**LG**-**1** and overcame the drawbacks of polarity and charge of [^18^F]**LN** [29,35], consequently generating better tumor imaging than [^18^F]**LN**, while the more lipophilicity and weaker PD-L1 binding affinity contributed to the poorer PET images than [^18^F]**LG**-**1**. In spite of some deficiencies, [^18^F]**LP**-**F** could still image PD-L1 in different tumors. It provided a new strategy for design of PD-L1 radiotracers. Introduction of hydroxyl groups to the biarylmethyl aryl ether scaffold in the solvent interaction region is another feasible way of modification to increase the solubility and the PD-L1 affinity of small-molecule PET agent, which could increase more accumulation and more retention time in PD-L1^+^ tumors and accelerate clearance from non-target organs. Construction of satisfactory PD-L1 tracers needs to coordinate all the factors, including lipophilicity, polarity, stability and radiolabeling methods to mediate the high binding affinity, which still remains a big challenge to fit within the routine clinical work. Such small-molecule PET agent could visualize PD-L1^+^ tumor rapidly within 2 h, which would lessen the burden on patients in terms of the total time of examination and the dose of radiation absorbed. The diagnostic reports could provide significant clinical guidance for patient stratification and recommend that patients whose tumors are PD-L1^+^ should receive immunotherapy. 

## 4. Materials and Methods

### 4.1. Chemistry

#### 4.1.1. Synthesis of LP1

A mixture of compound **L3** (500.0 mg, 0.95 mmol), piperazine (331.0 mg, 3.85 mmol) and acetic acid (239.0 mg, 3.98 mmol) in DMF (5 mL) was stirred at 45 °C for 4.5 h. After that, the flask was placed in an ice bath. Sodium cyanoborohydride (NaBH_3_CN, 241.1 mg, 3.83 mmol) was added to the flask and then the mixture was stirred for 24 h at 25 °C. The mixture was decanted into 80 mL water and extracted with DCM (40 mL × 3). The organic phase was collected, dried and evaporated. The residue was subjected to silica column chromatography with DCM/MeOH (15:1) as eluents to give compound **LP1** (128.6 mg, 22.7%) as a white solid. ^1^H NMR (400 MHz, DMSO-*d*_6_) *δ* 8.69 (bro, 1H), 7.98 (s, 1H), 7.85 (d, *J* = 8.0 Hz, 2H), 7.64 (t, *J* = 7.8 Hz, 1H), 7.46 (s, 1H), 7.45 (d, *J* = 7.8 Hz, 1H), 7.25 (t, *J* = 7.5 Hz, 1H), 7.19 (dd, *J* = 7.8, 1.0 Hz, 1H), 7.17 (s, 1H), 6.93 (d, *J* = 8.2 Hz, 1H), 6.77 (d, *J* = 2.0 Hz, 1H), 6.75 (dd, *J* = 8.2, 2.0 Hz, 1H), 5.31 (s, 2H), 5.26 (s, 2H), 4.28 (s, 4H), 3.50 (s, 2H, overlapped), 3.18 (s, 4H), 2.50 (s, 4H, overlapped), 2.24 (s, 3H). ESI-MS (*m/z*): 596.42 [M + H]^+^.

#### 4.1.2. Synthesis of LP2

A solution of compound **LP1** (247.0 mg, 0.415 mmol) in acetonitrile (12.5 mL) was charged with potassium carbonate (581.0 mg, 4.21 mmol) and ((oxybis(ethane-2,1-diyl))bis(oxy))bis(ethane-2,1-diyl) bis(4-methylbenzenesulfonate) (**TsO-PEG4-OTs**, 675.0 mg, 1.34 mmol). The resulting solution was heated to 85 °C for approximately 16 h and then cooled. After centrifugation for 10 min the solvent was evaporated, and the residue was purified by silica gel chromatography with TCM/ MeOH (20:1) as eluents to give compound **LP2** (130.2 mg, 33.9%) as colorless thick oil. ESI-MS (*m/z*): 926.58 [M + H]^+^.

#### 4.1.3. Synthesis of TsO-PEG4-F

A solution of compound **TsO-PEG4-OTs** (205.0 mg, 0.408 mmol) in THF (5 mL) was mixed with tetrabutylammonium fluoride (TBAF, 216 mg, 0.828 mmol) and was heated to 80 °C for approximately 20 min. After being cooled, the solvent was evaporated and purified by silica gel chromatography with Hex/EA (1:1) as eluents. The product was further purified by the semi-preparative HPLC system to obtain **TsO-PEG4-F** as colorless oily liquid (71.4 mg, 50.0%).^1^H NMR (400 MHz, CDCl_3_) *δ* 7.79 (d, *J* = 8.3 Hz, 2H), 7.33 (d, *J* = 8.0 Hz, 2H), 4.55 (dt, ^2^*J*_H-F_ = 47.7 Hz, ^3^*J*_H-H_ = 4.2 Hz, 2H), 4.15 (t, *J* = 4.8 Hz, 2H), 3.77 (t, *J* = 4.2 Hz, 1H), 3.70–3.62 (m, 7H), 3.59 (s, 4H), 2,44 (s, 3H). ESI-MS (*m/z*): 373.30 [M + Na]^+^.

#### 4.1.4. Synthesis of LP-F

Compounds **LP1** (15 mg, 0.0252 mmol) and **TsO**-**PEG4**-**F** (13.3 mg, 0.038 mmol) were stirred with potassium carbonate (34.5 mg, 0.252 mmol) in 5 mL ACN at 85 °C for 24 h. After the reaction, the solution was cooled, filtered and evaporated. The residue was purified by silica column chromatography with TCM/MeOH (15:1) as eluents to obtain product **LP**-**F** as white solid (13.3 mg, 68.2%).^1^H NMR (500 MHz, DMSO-*d*_6_) *δ* 7.95 (s, 1H), 7.84 (d, *J* = 7.8 Hz, 1H), 7.82 (d, *J* = 7.8 Hz, 1H), 7.63 (t, *J* = 7.8 Hz, 1H), 7.45 (d, *J* = 6.7 Hz, 1H), 7.29 (s, 1H), 7.25 (t, *J* = 7.6 Hz, 1H), 7.18 (dd, *J* = 7.7,1.1 Hz, 1H), 7.08 (s, 1H), 6.93 (d, *J* = 8.2 Hz, 1H), 6.78 (d, *J* = 2.1 Hz, 1H), 6.75 (dd, *J* = 8.2, 2.1 Hz, 1H), 5.28 (s, 2H), 5.22 (s, 2H), 4.50 (dt, ^2^*J*_H-F_ = 48.0 Hz, ^3^*J*_H-H_ = 4.1 Hz, 2H), 4.28 (s, 4H), 3.63 (dt, ^3^*J*_H-F_ = 31.2 Hz, ^3^*J*_H-H_ = 4.1 Hz, 2H), 3.53–3.47 (m, 10H), 3.42 (s, 2H), 2.46–2.30 (m, 10H), 2.24 (s, 3H). ^13^C NMR (126 MHz, DMSO-*d*_6_) *δ* 155.77, 153.22, 142.95, 142.50, 141.63, 138.79, 135.05, 134.37, 134.03, 132.13, 131.63, 130.81, 130.71, 129.72, 129.61, 127.59, 125.45, 122.10, 120.12, 118.67, 117.70, 116.78, 112.83, 111.47, 100.73, 83.00 (d, ^1^*J*_C-F_ = 169.3 Hz), 69.82, 69.75, 69.65 (d, ^2^*J*_C-F_ = 18.9 Hz), 69.63, 68.78, 68.24, 64.10, 64.08, 57.17, 55.12, 53.18, 52.66, 15.86. ^19^F NMR (376 MHz, CDCl_3_) *δ* -222.7 (tt, ^2^*J*_F-H_ = 47.5 Hz, ^3^*J*_F-H_ = 29.9 Hz). ESI-MS (*m/z*): 774.57 [M + H]^+^.

### 4.2. Radiosynthesis of PD-L1 PET Imaging Agent [^18^F]LP-F

[^18^F]Fluoride was obtained by ^18^O(p, n)^18^F nuclear reaction through a medical cyclotron (HM7, Sumitomo Heavy Industries) and trapped on an anion-exchange Sep-Pak light QMA column, which had been activated by NaHCO_3_ aqueous solution (0.5 M, 10 mL) and distilled water (10 mL). The QMA cartridge with [^18^F]fluoride was eluted with a solution of Kryptofix 2.2.2 and K_2_CO_3_ in 1.5 mL of acetonitrile/water. The solution was evaporated using a stream of nitrogen at 110 °C and coevaporated to dryness with ACN (2.0 mL) to remove water. The labeling precursor, compound **LP2** (approximately 3.6 mg), dissolved in DMSO (0.6~0.7 mL), was added and reacted with ^18^F^-^ (approximately 11.1 GBq) at 110 °C for 30 min (Figure 1A). The ^18^F-labeled crude product was analyzed by radio-HPLC by comparing the retention time (t_R_) of [^18^F]**LP**-**F** with that of the non-radioactive probe **LP**-**F**. The [^18^F]**LP**-**F** was subjected to semi-preparative HPLC, diluted in water, concentrated on C18 light Sep-Pak cartridge, washed by water and eluted by ethanol (0.8~1.0 mL).

### 4.3. Molar Activity Test

The UV spectrum of compound **LP**-**F** was drawn by UV spectrophotometer to aquire the λ_max_ of **LP**-**F**. Concentrations of **LP**-**F** were subjected to UV-HPLC at λ_max_ (286 nm) to establish calibration curve using peak areas of corresponding concentrations of **LP**-**F** and afforded linear regression equation through peak areas and concentrations. After injection of purified [^18^F]**LP**-**F**, the UV peak area at 286 nm of purified [^18^F]**LP**-**F** was used to calculate the injection concentration according to above linear regression equation. The molar activity was calculated based on Eq.

Molar activity = Injection radioactivity/Amount of substance injected

### 4.4. In Vitro Stability Assay

In order to investigate the in vitro stability of the radioactive probe, [^18^F]**LP**-**F** (~14.8 MBq, 40 μL) was incubated in PBS (pH 7.4, 360 μL) at 37 °C for 4 h. [^18^F]**LP**-**F** (~14.8 MBq, 40 μL) was also mixed with mouse serum (360 μL) and maintained at 37 °C for 4 h. Such PBS solution was subjected to stability analysis using radio-HPLC at 0, 1, 2 and 4 h. A 30 μL sample from the above mouse serum after protein precipitation with 30 μL acetonitrile was also subjected to stability analysis at 0, 1, 2 and 4 h.

### 4.5. Partition Coefficient Test

Amounts of 0.37 MBq [^18^F]**LP**-**F**, 1000 μL water and 1000 μL 1-octanol were mixed in a tube. The mixture was thoroughly shaken for 5 min. After centrifugation, 500 μL water was withdrawn from water phase and its radioactivity was measured by a gamma counter. An equal volume of 1-octanol was withdrawn from the organic phase and measured. The partition coefficient (Log *P*) was calculated based on Eq.

Log *P* = Lg(Concentration of [^18^F]**LP**-**F** in 1-octanol/Concentration of [^18^F]**LP**-**F** in water)

Then, 500 μL water and 500 μL 1-octanol were added to the above tube to replenish the water and the organic phase. The two-phase system was shaken again. Water and the organic phase were sampled and measured and Log *P* was calculated again. Repeat two more times.

### 4.6. Cell Lines and Animal Models

Human melanoma cell line A375 (PD-L1^-^) and human PD-L1 gene-transfected A375 cells (A375-hPD-L1, PD-L1^+^) were cultured according to previous work.

Female BALB/c nude mice at age of 5~7 weeks were inoculated subcutaneously with 1 × 10^6^ A375-hPD-L1 cells in saline at the left flank and 1 × 10^6^ A375 cells at the right flank in the same mouse in order to eliminate individual differences. Animal study can be performed in accordance with the principles laid out by the ethical committee of Jiangsu Institute of Nuclear Medicine.

### 4.7. Cell Viability Assay

Each well was seeded with 1 × 10^4^ cancer cells in 96-well plates. **LP**-**F** dissolved in DMEM with gradient concentration (0, 12.5, 25, 50 and 100 μM) was added to each well at triple parallels, respectively, and incubated for 24 h. Then, MTT (5 mg/mL, 20 μL/well) was added and incubated for 4 h. 150 μL DMSO was added each well and shaken vigorously for 10 min to dissolve MTT crystallization. The absorbance of each sample was detected at 490 nm by ELISA, and percent cell viability was calculated based on Eq.:

Percentage = sample well/reference well × 100%

### 4.8. Cell Uptake

A375 cells and A375-hPD-L1 cells (1 × 10^6^) were added to each tube, separately. [^18^F]**LP**-**F** (37 kBq/tube) diluted in DMEM was added and incubated at 37 °C for different periods (30, 60, 120, and 240 min). PD-L1^+^ cells were incubated with 100 μL 25 μM **LP**-**F** for 30 min ahead to block PD-L1^+^ cells and then incubated with [^18^F]**LP**-**F** (37 kBq/tube) at same conditions. After incubation and wash, the cells’ radioactivity was measured.

### 4.9. The Saturation Binding Assay

PD-L1^+^ cells (2 × 10^5^) were added to each tube and incubated with the gradient of concentration of [^18^F]**LP**-**F** dissolved in DMEM (0 to 128 nM) at 37 °C for 1 h. After incubation, the cells were centrifuged and washed by PBS twice and then were measured for radioactivity by a γ counter as total bound value. For the non-specific bound value, PD-L1^+^ cells (2 × 10^5^) were added to each tube and incubated with **LP**-**F** (50 μM) dissolved in DMEM for half an hour in advance and then incubated with above concentrations of [^18^F]**LP**-**F**. After centrifugation and wash, the radioactivity was detected. The specific binding value is obtained by subtracting non-specific bound value from total bound value. The equilibrium dissociation constant (K_d_) was calculated by non-linear fit in a one-site model through specific binding values and concentrations of [^18^F]**LP**-**F**.

### 4.10. Micro-PET Imaging

A375 (right)/A375-hPD-L1 (left) bilateral tumor-bearing mice (*n* = 3) were anesthetized and then intravenously injected with [^18^F] **LP**-**F** (~7.4 MBq) dissolved in saline (200 μL). Dynamic scanning was performed for one hour. ASIPro software (Siemens) was applied to process the PET images. Dynamic images were reconstructed in OSEM3D/MAP algorithm and split into twelve frames. Quantification and analysis of region of interest (ROI) in tumors and organs were carried out.

### 4.11. Pharmacokinetics

Female BALB/c normal mice (*n* = 3) were injected (i.v.) with [^18^F] **LP**-**F** (~7.4 MBq, 200 μL) via tail vein and the tails were cut off (p.i.) to obtain the blood samples at setting time points. Meanwhile, 25 μL [^18^F] **LP**-**F** (~0.925 MBq) was set as reference. After weighing, radioactivity of each blood sample was tested immediately. The drug concentration–time curve was draw. DAS 2.1 software was employed to calculate the pharmacokinetic parameters. A compartment model was used to analyze the distribution and clearance process of the radioactive probe [^18^F] **LP**-**F** in vivo to demonstrate the results of PET and the biocompatibility of the radiotracer.

### 4.12. Statistical Analysis

Data were presented as the mean ± SD. The differences among groups were analyzed by two-tailed Student’s *t*-test indicated as * *p* < 0.05, ** *p* < 0.01 and *** *p* < 0.001.

## 5. Conclusions

In summary, a small molecule, [^18^F]**LP-F,** radiolabeled with ^18^F was explored as a novel PET imaging radiotracer for assessment of PD-L1 expression in tumors. [^18^F]**LP**-**F** showed high stability in vitro and in vivo. The moderate affinity to PD-L1 made it selectively accumulate in PD-L1 overexpression tumor cells. The PET imaging studies demonstrated its ability to measure PD-L1 expression in vivo. This study provided opportunities to explore the design of small-molecule PET tracers for assessing PD-L1 expression in tumors.

## Data Availability

Data are contained within the article and Supplementary Material.

## Supplementary Materials

The following supporting information can be downloaded at: https://www.mdpi.com/article/10.3390/ph16020213/s1, Figure S1: HPLC characterization of compound **LP1**; Figure S2: ESI-MS spectrum of compound **LP1**; Figure S3: ^1^H NMR spectrum of compound **LP1** in DMSO-*d*_6_; Figure S4: HPLC characterization of compound **LP2**; Figure S5: ESI-MS spectrum of compound **LP2**; Figure S6: HPLC characterization of compound **TsO-PEG4-F**; Figure S7: ESI-MS spectrum of compound **TsO-PEG4-F**; Figure S8: ^1^H NMR spectrum of compound **TsO-PEG4-F** in CDCl_3_; Figure S9: HPLC characterization of compound **LP**-**F**; Figure S10: ESI-MS spectrum of compound **LP**-**F**; Figure S11: ^1^H NMR spectrum of compound **LP**-**F** in DMSO-*d*_6_; Figure S12: ^13^C NMR spectrum of compound **LP**-**F** in DMSO-*d*_6_; Figure S13: ^19^F NMR spectrum of compound **LP**-**F** in CDCl_3_; Figure S14: The UV spectrum of compound **LP**-**F** in ACN and H_2_O; Figure S15: Calibration curve of compound **LP**-**F**; Figure S16: Radiosynthesis of [^18^F]**LN** and [^18^F]**LG**-**1** and specifications of [^18^F]**LN,** [^18^F]**LG**-**1** and [^18^F]**LP-F.** (A) Radiosynthesis of [^18^F]**LN** via one-step ^18^F-^19^F isotope exchange and Radiosynthesis of [^18^F]**LG**-**1** via oxime formation. (B) Comparison of biological parameters between [^18^F]**LN**, [^18^F]**LG**-**1** and [^18^F]**LP**-**F**.Table S1: HPLC conditions for analysis of compounds; Table S2: The distribution of [^18^F]**LP**-**F** in 1-octanol phase and water phase and its partition coefficient (Log *P* = 2.18 ± 0.16).

## Abbreviations

DMEM	Dulbecco’s modified eagle medium
ESI-MS	Electro Spray Ionization-Mass Spectroscopy
HPLC	High performance liquid chromatography
IHC	Immunohistochemistry
MTT	3-(4,5-dimethyl-2-thiazolyl)-2,5-diphenyl-2-tetrazolium bromide
NMR	Nuclear Magnetic Resonance
OTs	P-Toluenesulfonyloxy
PBS	Phosphate buffer saline
PD-1	Programmed cell death protein 1
PD-L1	Programmed death ligand 1
PEG	Poly(ethylene glycol)
PET	Positron emission tomography
ROI	Region of interest
T/M	Tumor to muscle ratio
