# Supplementary material for: Design, Synthesis, and Biological Evaluation of a Small-Molecule PET Agent for Imaging PD-L1 Expression
Source: Pharmaceuticals (Basel). 2023 Jan 30;16(2):213. doi: 10.3390/ph16020213 (PMC9968138; doi:10.3390/ph16020213)
Supplement: Supplementary file 1 [file pharmaceuticals-16-00213-s001.zip › pharmaceuticals-2099446-supplementary.docx]

***Supporting information***

Design, Synthesis, and Biological Evaluation of a Small-molecule PET Agent for Imaging PD-L1 Expression

Liang Xu ^1,2^, Lixia Zhang^1,2^, Beibei Liang^1,2^, Shiyu Zhu^2^, Gaochao Lv^2^, Ling Qiu^1,2,*^ and Jianguo Lin^1,2,^*

^1 School of Basic Medical Sciences, Wenzhou Medical University, Wenzhou, 325035, China; 13677959323@163.com (L.X.); zhanglixia1017@126.com (L.Z.); 15908119793@163.com (B.L.)^

^2 NHC Key Laboratory of Nuclear Medicine, Jiangsu Key Laboratory of Molecular Nuclear Medicine, Jiangsu Institute of Nuclear Medicine, Wuxi, 214063, China; zhushiyurainbow@163.com (S.Z.); lvgaochao@jsinm.org (G.L.)^

**^*^** ^Correspondence: qiuling@jsinm.org (L.Q.); linjianguo@jsinm.org (J.L.); Tel.: +86-0510-8551-4482-3505 (J.L.)^

**Characterization**


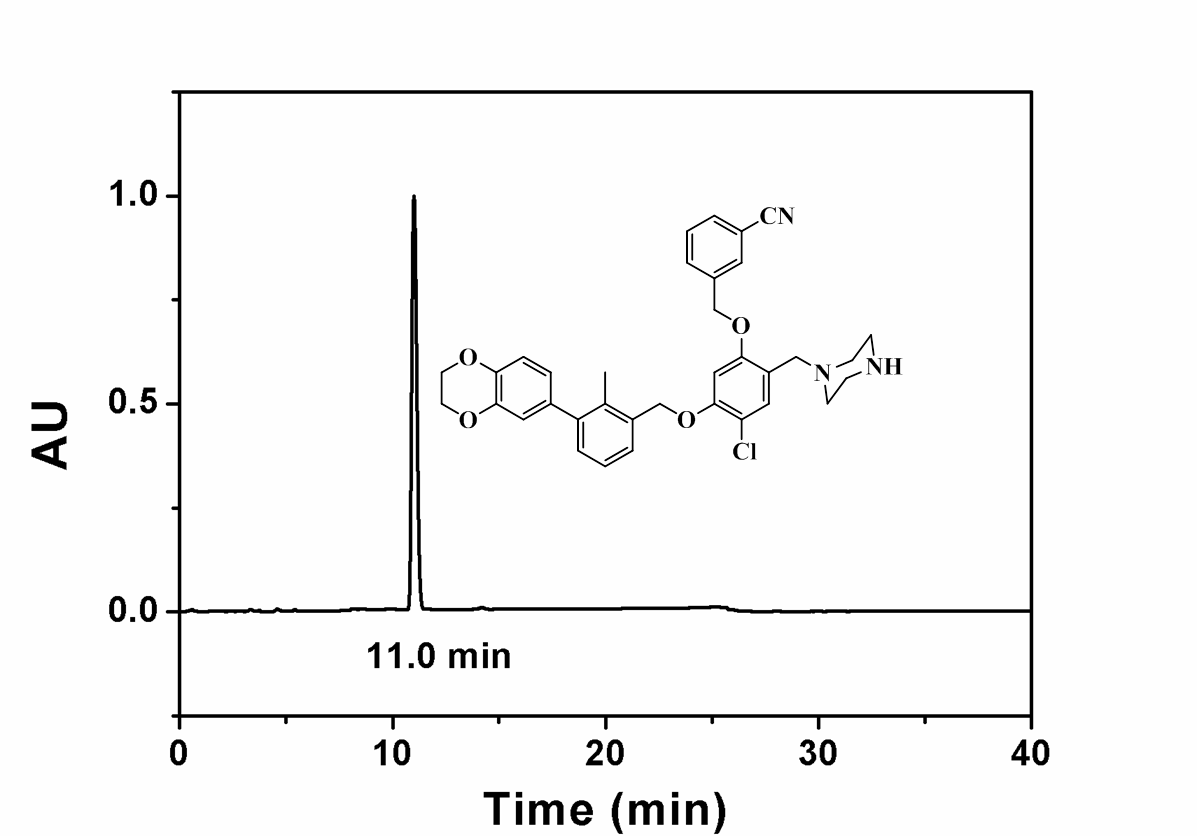


**Figure S1.** HPLC characterization of compound **LP1**.


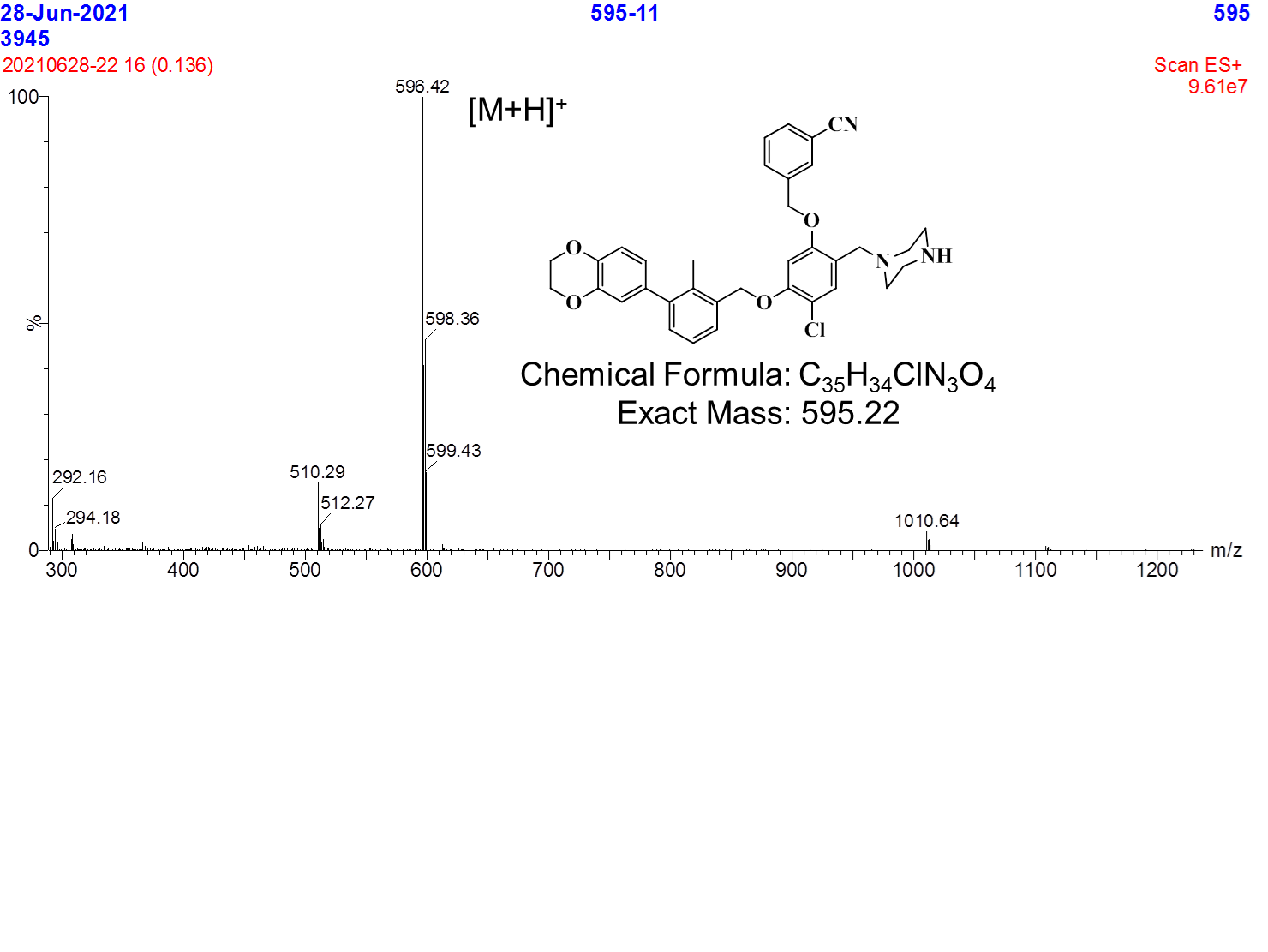


**Figure S2.** ESI-MS spectrum of compound **LP1**.

**Figure S3.** ^1^H NMR spectrum of compound **LP1** in DMSO-*d*_6._

**
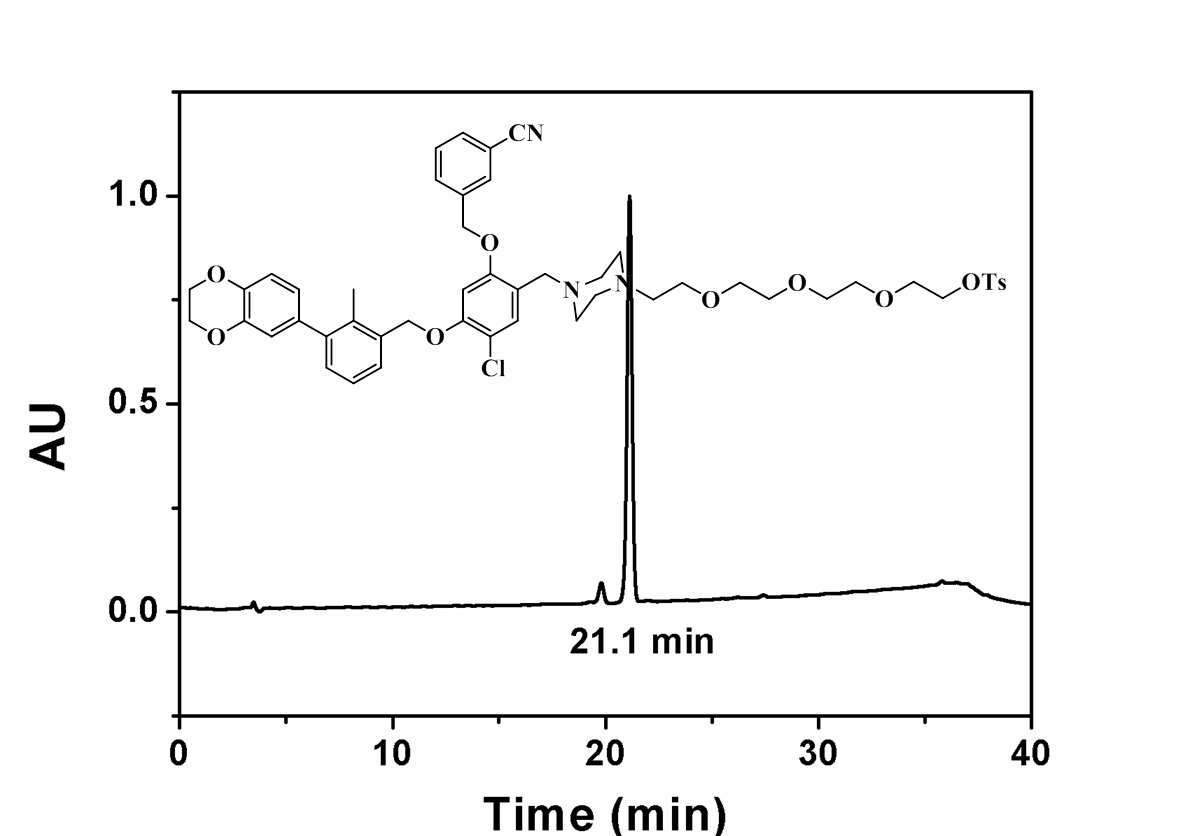
**

**Figure S4.** HPLC characterization of compound **LP2**.

**
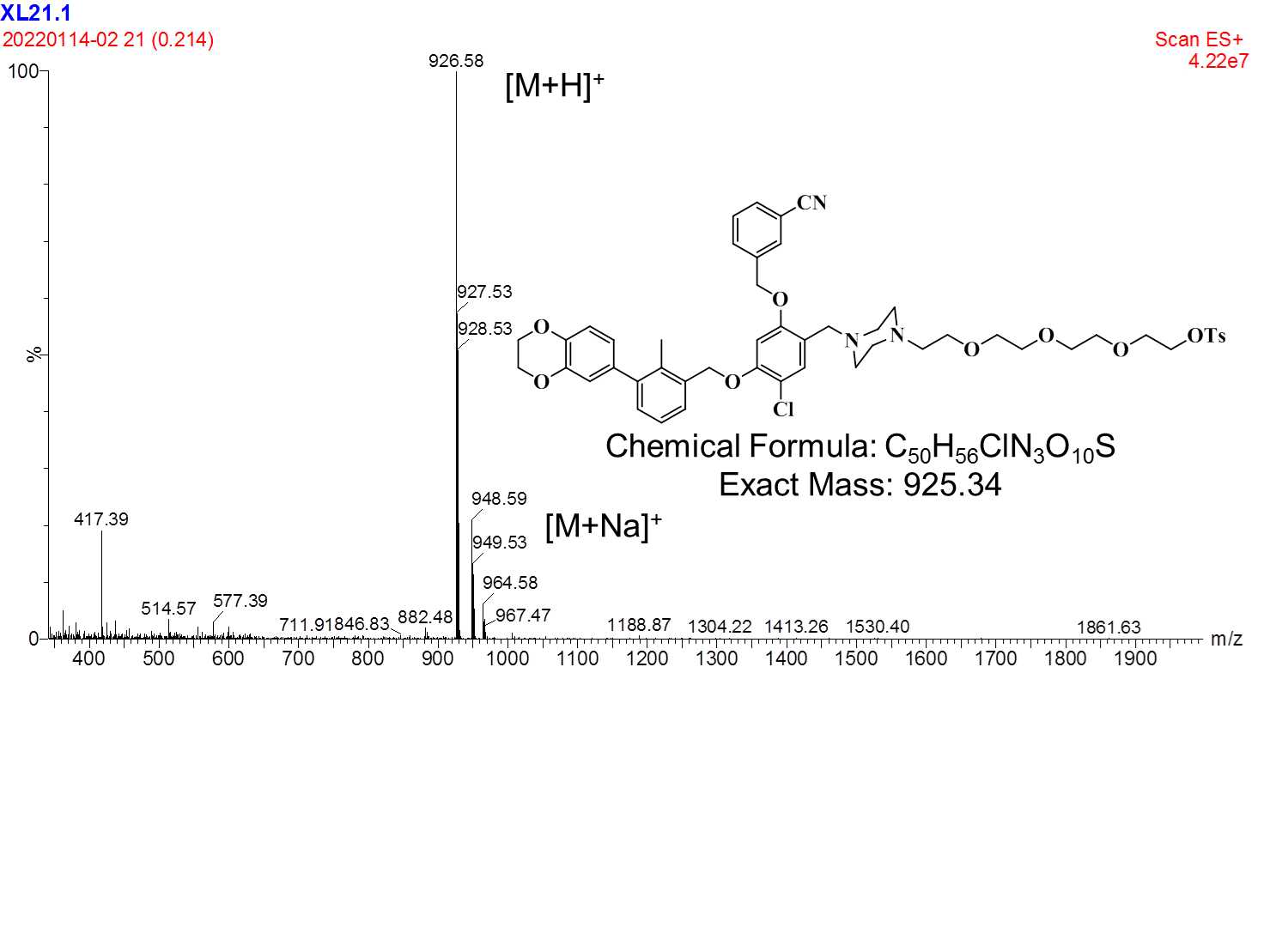
**

**Figure S5.** ESI-MS spectrum of compound **LP2**.


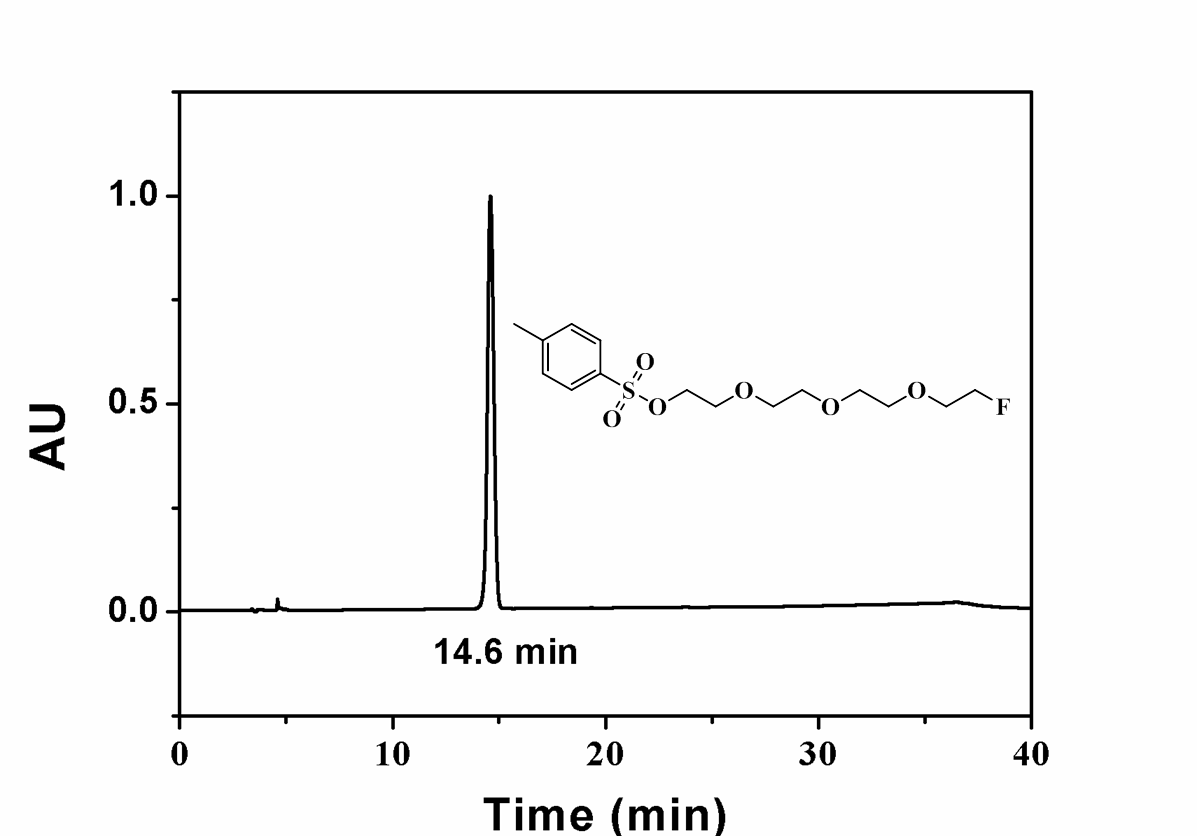


**Figure S6.** HPLC characterization of compound **TsO-PEG4-F.**


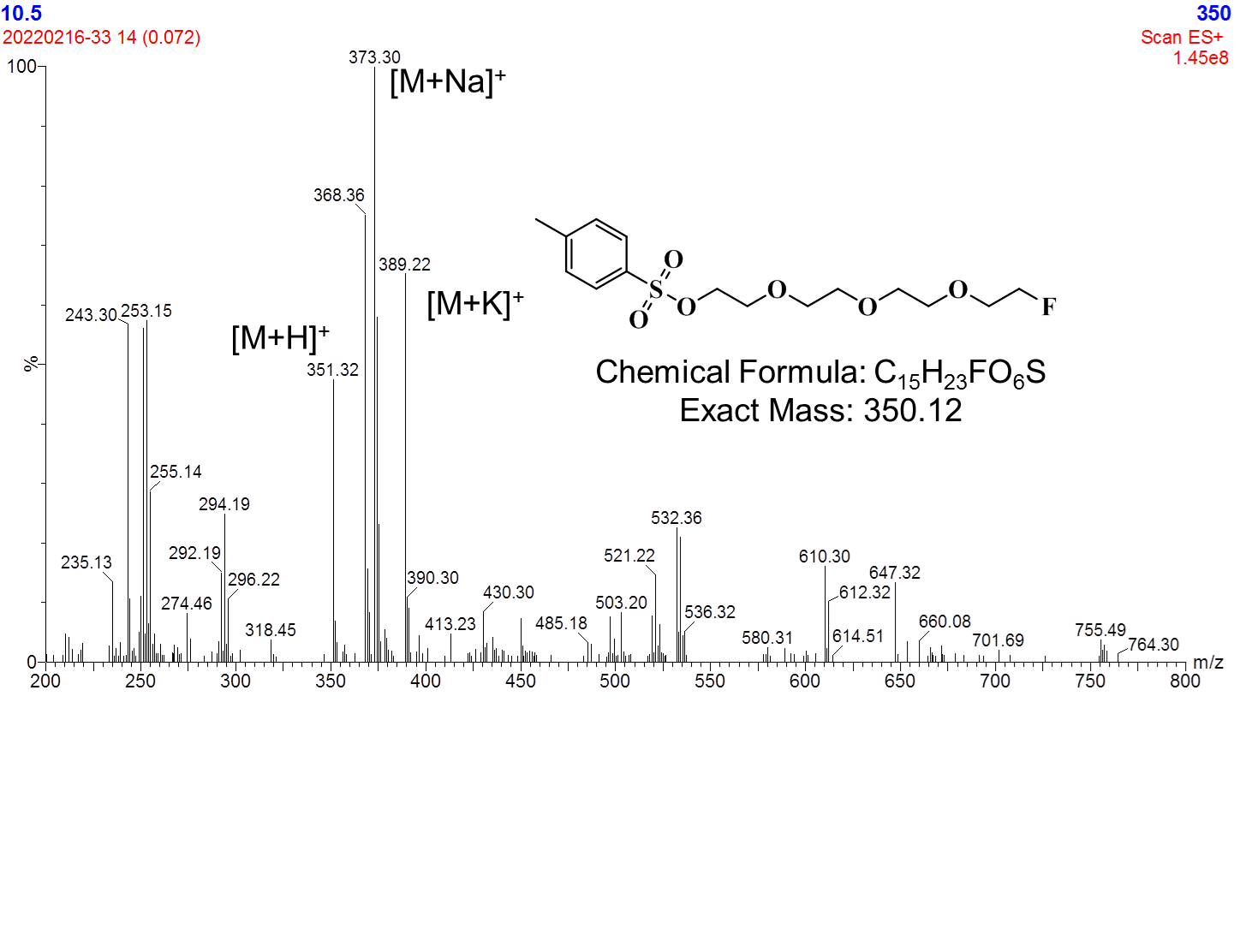


**Figure S7.** ESI-MS spectrum of compound **TsO-PEG4-F.**

**Figure S8.** ^1^H NMR spectrum of compound **TsO-PEG4-F** in CDCl_3._


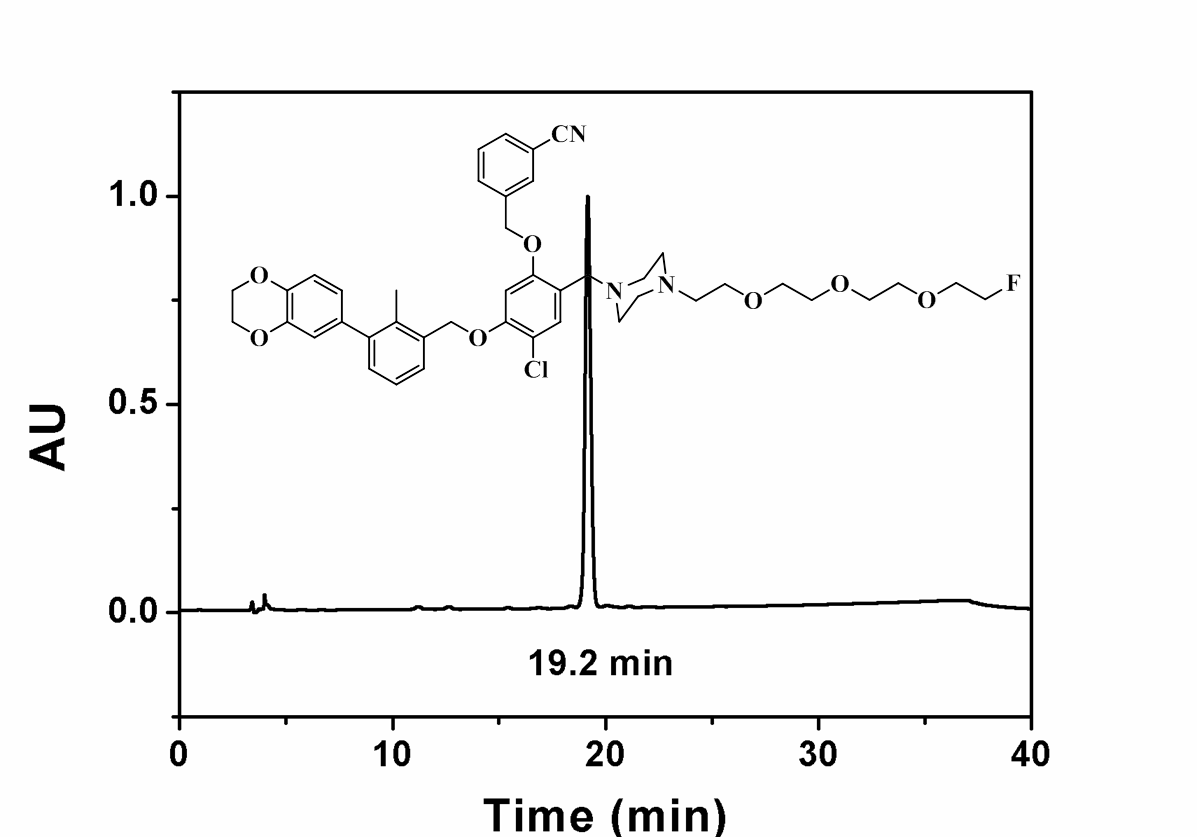


**Figure S9.** HPLC characterization of compound **LP-F**.

**
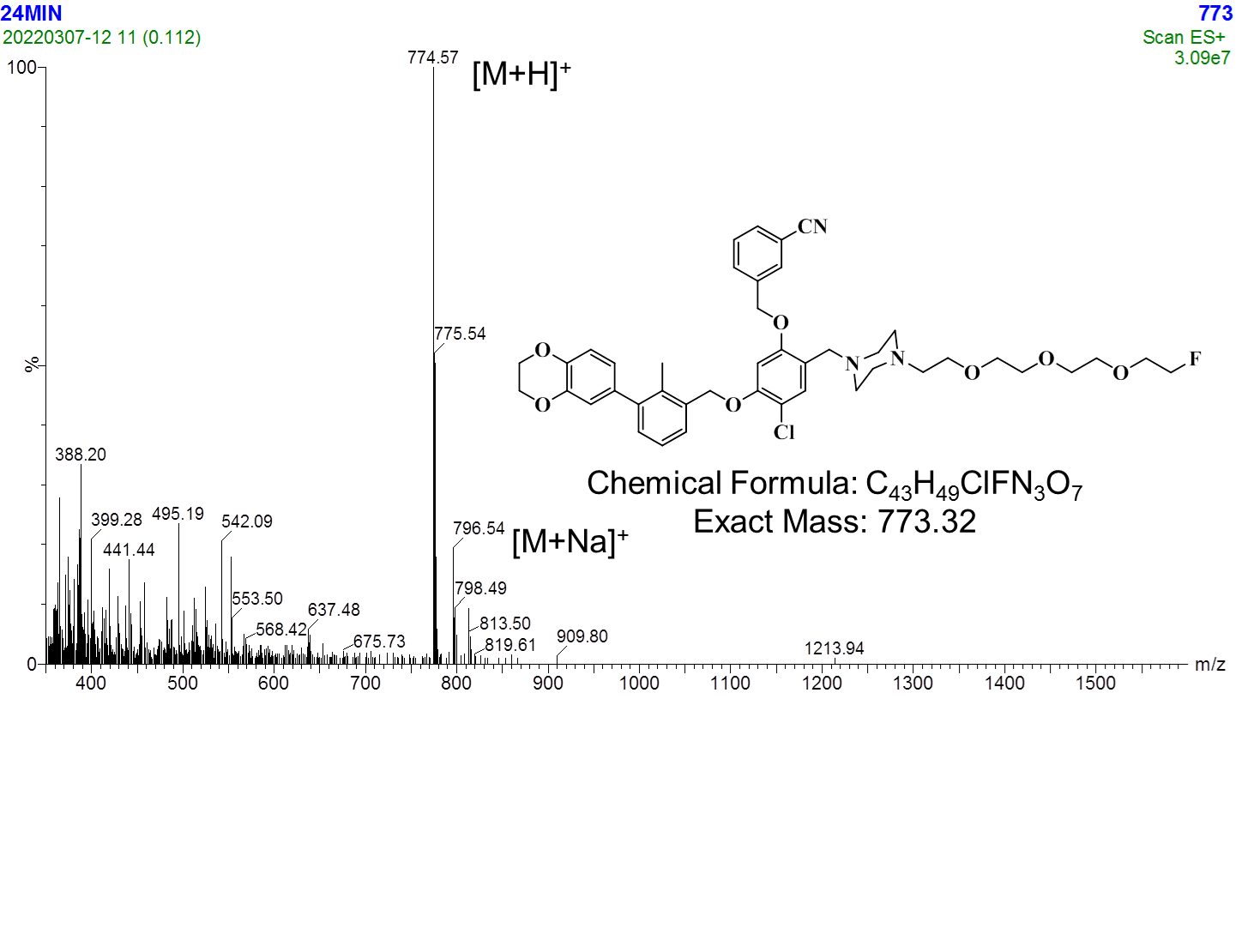
**

**Figure S10.** ESI-MS spectrum of compound **LP-F**.

**Figure S11.** ^1^H NMR spectrum of compound **LP-F** in DMSO-*d*_6._

**Figure S12.** ^13^C NMR spectrum of compound **LP-F** in DMSO-*d*_6._

**Figure S13.** ^19^F NMR spectrum of compound **LP-F** in CDCl_3._

**Figure S14.** The UV spectrum of compound **LP-F** in ACN and H_2_O.

**Calibration curve**

**Figure S15.** Calibration curve of compound **LP-F.**

**Comparison between radiotracers**

**
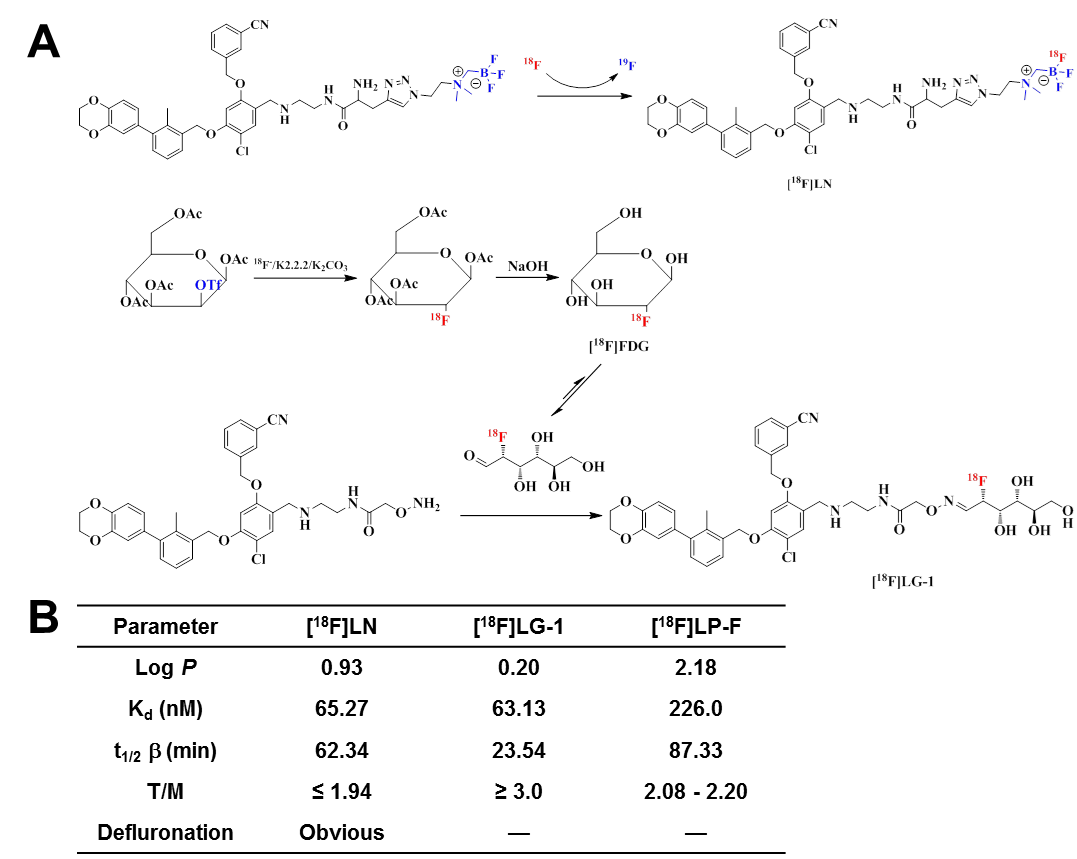
**

**Figure S16.** Radiosynthesis of [^18^F]**LN** and [^18^F]**LG-1** and specifications of [^18^F]**LN**, [^18^F]**LG-1** and [^18^F]**LP-F**. (A) Radiosynthesis of [^18^F]**LN** via one-step ^18^F-^19^F isotope exchange and Radiosynthesis of [^18^F]**LG-1** via oxime formation. (B) Comparison of biological parameters between [^18^F]**LN**, [^18^F]**LG-1** and [^18^F]**LP-F**.

**HPLC conditions**

**Table S1.** HPLC conditions for analysis of compounds

| Time/min | Flow (mL/min) | H_2_O (0.1% TFA)% | ACN (0.1% TFA)% |
| --- | --- | --- | --- |
| Initial | 1 | 80 | 20 |
| 3 | 1 | 80 | 20 |
| 32 | 1 | 5 | 95 |
| 40 | 1 | 80 | 20 |

**Partition coefficient test**

**Table S2.** The distribution of [^18^F]**LP-F** in 1-octanol phase and water phase and its partition coefficient (Log *P* = 2.18 ± 0.16)

| Trial | CPM in water phase | CPM in 1-octanol phase | Log *P* |
| --- | --- | --- | --- |
| 1 | 20923 | 4870338 | 2.37 |
| 2 | 20556 | 2477227 | 2.08 |
| 3 | 10565 | 1343973 | 2.10 |
